# Salinity stress improves antioxidant potential by modulating physio-biochemical responses in *Moringa oleifera* Lam.

**DOI:** 10.1038/s41598-023-29954-6

**Published:** 2023-02-18

**Authors:** Muhammad Azeem, Kulsoom Pirjan, Muhammad Qasim, Athar Mahmood, Talha Javed, Haji Muhammad, Shoujun Yang, Renjie Dong, Baber Ali, Mehdi Rahimi

**Affiliations:** 1 Biosaline Research Laboratories, Department of Botany, University of Karachi, Karachi, 75270 Pakistan; 2 Dr. Muhammad Ajmal Khan Institute of Sustainable Halophyte Utilization, University of Karachi, Karachi, Pakistan; 3 Department of Agronomy, University of Agriculture Faisalabad, Faisalabad, 38040 Pakistan; 4 College of Agriculture, Fujian Agriculture and Forestry University, Fuzhou, 350002 China; 5 Department of Chemistry, Federal Urdu University of Arts, Sciences and Technology, Gulshan-E-Iqbal Campus, Karachi, 75300 Pakistan; 6 Yantai Institute, China Agricultural University, Yantai, 264670 Shandong Province China; 7 Department of Plant Sciences, Quaid-i-Azam University, Islamabad, 45320 Pakistan; 8 Department of Biotechnology, Institute of Science and High Technology and Environmental Sciences, Graduate University of Advanced Technology, Kerman, Iran

**Keywords:** Ecology, Plant sciences

## Abstract

*Moringa oleifera* Lam*.* is a common edible plant, famous for several nutritional and therapeutic benefits. This study investigates the salt -induced modulations in plant growth, physio-biochemical responses, and antioxidant performance of *M. oleifera* grown under 0, 50, and 100 mM NaCl concentrations. Results showed that the plant effectively managed moderate salinity (50 mM NaCl) by maintaining succulence, weight ratios, and biomass allocation patterns of both shoot and root with minimal reduction in dry biomass. However, high salinity (100 mM NaCl) remarkably declined all growth parameters. The plant accumulated more Na^+^ and Cl^−^, while less K^+^ under salinity as compared to the control. Consequently, osmotic potentials of both root and leaf decreased under salinity, which was corroborated by the high amount of proline and soluble sugars. Increased level of H_2_O_2_ with significantly unchanged membrane fluidity indicating its role in perceiving and managing stress at moderate salinity. In addition, increased activities of superoxide dismutase, and catalase, with increased glutathione and flavonoid contents suggest an integrated participation of both enzymatic and non-enzymatic antioxidant components in regulating ROS. On the other hand, high salinity caused an outburst of ROS indicated by high H_2_O_2_, MDA, and electrolyte leakage. As a response, moringa drastically increased the activities of all antioxidant enzymes and contents of antioxidant molecules including ascorbic acid, glutathione, total phenols, and flavonoids with high radical scavenging and reducing power capacities. However, a considerable amount of energy was used in such management resulting in a significant growth reduction at 100 mM NaCl. This study suggests that moringa effectively resisted moderate salinity by modulating physio-biochemical attributes and effectively managing ion toxicity and oxidative stress. Salt stress also enhanced the medicinal potentials of moringa by increasing the contents of antioxidant compounds including ascorbic acid, glutathione, total phenols, and flavonoids and their resulting activities. It can be grown on degraded/ saline lands and biomass of this plant can be used for edible and medicinal purposes, besides providing other benefits in a global climate change scenario.

## Introduction

Salt tolerant plants acquire various physiological and metabolic adaptations to avoid specific ion toxicity, osmotic shock^1–3^, nutrient imbalance^4^ and oxidative damage^5–7^. Increase in root and/ or shoot biomass also helps in regulating Na^+^ entry to the xylem or to decrease its transport to the shoot^8,9^ while excluding and avoiding toxic ions during the process^10^. Active nutrients, in this case, protects photosynthetic pigments and ensure photosynthetic efficiency during the process^11–15^. The concentration and composition of leaf pigments may differ based on the level of tolerance of a species^16,17^. Disturbance in ion homeostasis may leads to ion toxicity, which obstructs the photosynthetic process by degrading the photosynthetic pigments or affecting their biosynthesis^18–21^. In addition, declining photosynthesis could be due to the salt induced reduction of the biochemical potential to fix CO_2_ as well as up-surging of respiration, resulting an alteration in biomass provision patterns^22^.

Under salt stress, excessive energy headed for molecular oxygen activates the oxygen poisoning by over-production of singlet oxygen, superoxide ion, hydrogen peroxide and other free oxygen radicals^23–26^. Such free radicals are damaging for proteins, lipids, nucleic acid and other macromolecules^27–29^. Even then, these radicals initiate a chain reaction, which leads to the dilapidation of cellular and sub-cellular membranes including mitochondria, chloroplast, and other organelles^30–32^. Similarly, during the photo-respiration process, corrosion proceeds through H_2_O_2_, where oxidation of glycolate occurs^33,34^. Plant employs an efficient antioxidant system to overcome slat induced oxidative stress^35,36^. This includes antioxidant enzymes like superoxide dismutase (SOD), catalase (CAT), ascorbate peroxidase (APX), polyphenol oxidase (POX) and others. Furthermore, non-enzymatic antioxidants such as glutathione, ascorbic acid, polyphenols, flavonoids, tocopherol, anthocyanins, carotenoids, and others also play a significant role in detoxification of ROS. The SOD is considered as a first line of defense, which converts superoxide into H_2_O_2_. In further, CAT and APX converts H_2_O_2_ into H_2_O^37^. Glutathione peroxidase (GPX) also converts cytotoxic H_2_O_2_ into alcohols and H_2_O^38^. In further, GPX purifies lipid peroxidation products through catalytic process (Fenton reaction). Ascorbic acid act as an efficient antioxidant and used as the electron donor, even though the ascorbate–glutathione cycle decreases H_2_O_2_ to H_2_O using APX^39^. It also assists in the production of zeaxanthin during the xanthophyll cycle and tocopherol synthesis in different cellular compartments, which helps in heat dissipation mechanism under stress^17^. Besides enzymatic antioxidant system, secondary phytochemicals including phenols, flavonoids, anthocyanins, and tannins also strengthen the overall antioxidant performance of plant by direct quenching of damaging free radicals^40–42^. Among these, polyphenols and flavonoids are powerful antioxidant molecules. These active electron donating compounds halt the oxidative chain reaction and protect macromolecules and membranes under stress^43,44^. In further, these compounds possess several health promoting and disease preventing effects, hence used for medicinal purposes at local and industrial scale.

*Moringa oleifera* Lam. is native to Pakistan and belongs to family Moringaceae. It is distributed throughout the country and exported to nearby states in the region. It is known as Horseradish tree or Drumstick, while in Pakistan it is locally called as Sohanjna. This small deciduous tree has the capability to grow on a variety of soil types and can endure abiotic stresses^45^. Different parts of this plant including leaves, buds, flowers, roots and pods are widely consumed as culinary as well as for medicinal purposes^46^. Moringa is a rich source of nutrients and minerals including beta-carotene, amino acids, vitamins, proteins, polyphenols, flavonoids and natural antioxidants. Scientific studies confirms the anti-inflammatory, anti-cancer, hepatoprotective, neuroprotective, and anti-aging properties of this plant^47^. This plant has a potential market in both food and pharmaceutical industries, besides local uses^46^ therefore, attracts the interest of local farmers for cultivating and harvesting it as a field crop. However, due to the rapid depletion of arable lands and freshwater resources especially in semiarid areas, it would become increasingly difficult for new crops to get their space. Interestingly, the resilience to climatic factors and environmental stressor allows moringa to grow under the conditions where conventional crops are failed to survive. However, the information about the tolerance mechanisms and the impact of applied stress on medicinal properties of this plant is not well documented. Therefore, this study investigates the effect of salinity on plant growth, biomass allocation, leaf pigments, ion accumulation, osmotic adjustment, antioxidant defense system and medicinal potential of *M. oleifera*.

## Materials and methods

### Experimental setup and plant growth

Moringa seeds were collected from trees growing in university of Karachi campus and vicinity and separated with the common physical qualities comparison. Healthy and uniform seeds were selected from a single seed lot and disinfected with 1% sodium hypochlorite solution. Seeds were sown in 200 × 100 mm pots containing sandy loam soil (Sand = 57.8%, Clay = 38.2%, Silt = 4%, water holding capacity = 30%) with 9:1 soil cow dung manure ratio, with six replicates in a completely randomized design. After 15 days of seed germination, healthy and uniform seedlings with similar vigour were picked for growth experiment. One seedling per pot was used for further experimentation. Seedlings were irrigated with half strength Hoagland’s solution containing 0, 50 and 100 mM NaCl solutions. To avoid the osmotic shock, salinity treatment was applied gradually at the rate of 25 and 50 mM NaCl per day for 50 and 100 mM NaCl treatments, respectively for two days to maintain the required concentration. The experiment was performed under ambient conditions in netted greenhouse with high temperature ranges from 32 to 36 °C, low temperature ranges from 24 to 28 °C, RH at 12 noon was 55–60%, photoperiod was 13.5 h, and PPFD at 12 noon was 1000–1200 μmol m^−2^ s^−1^. Plants were allowed to grow for 40 days and then harvested and analysed for their growth, eco-physiological responses and medicinal potential.

### Estimation of relative water content (RWC)

Relative water content was estimated by^48^ method. A completely developed leaf was removed from the 3rd node from top of the plant. After taking the fresh weight (W_1_), samples were placed in water for about 24 h to get a turgid weight (W_2_). Later on samples were oven-dried (48 h) to get dry weight (W_3_), and relative water content (RWC) was calculated by using the following equation:

$${\text{RWC }} = \, \left( {{\text{fresh }}\;{\text{weight }}{-}{\text{ dry }}\;{\text{weight}}} \right)/\left( {{\text{turgid}}\;{\text{ weight }}{-}{\text{ dry }}\;{\text{weight}}} \right) \, \times { 1}00$$

### Quantification of photosynthetic pigments

Fresh leaves samples (0.5 g) were extracted in 80% C_3_H_6_O (5 mL). Samples were then incubated for 20 min at 70 °C. Absorbance of extracts were taken at 662 nm and, 646 nm for chlorophylls, and 470 nm for carotenoids^49^.

### Determination of Na^+^ and K^+^

Oven dried samples (100 mg) were homogenized in H_2_O (10 mL) and extracted at boiling water bath for 2 h. Suitable dilution was prepared for Na^+^ and K^+^ and estimated through flame photometer (Jenway model 410).

### Measurement of leaf osmotic potential

Young and fresh leaves were excised from the top third node of each plant and kept under liquid nitrogen. Frozen samples were then crushed to get tissue sap that was further subjected to Osmette µ precision system to get leaf osmolality using Van’t Hoff equation^50^.

### Estimation of proline content

Dried plant samples (50 mg) were extracted with 3% sulphosalicylic acid (4 mL) to quantify proline in moringa leaves by ninhydrin reagent method^51^. Plant extract, ninhydrin reagent and glacial acetic were mixed in a similar proportion and boiled for 1 h. Toluene (2 mL) was added to the mixture and vortexed. The upper phase was separated, and absorbance was measured at 520 nm. Proline content was estimated against a standard curve using L-proline as standard.

### Estimation of total soluble sugars

Total soluble sugars were estimated by Anthrone method^52^. Anthrone (0.2 g) was added in 95% H_2_SO_4_ on ice bath with continuous stirring to prepare the anthrone reagent. Dried leaf samples (0.1 g) were added in test tubes containing 5 mL water and boil the samples to make hot water extract. Anthrone reagent (500 µL) was then added in a hot water extract (250 µL) and boiled for 11 min at 100 °C. After cooling, absorbance was recorded at 630 nm.

### Estimation of hydrogen peroxide, malondialdehyde and electrolyte leakage

Fresh leaf sample (0.5 g) was homogenized in 3% ice-cold TCA (5 mL) and centrifuged (12,000 rpm at 4 °C) for 20 min. Supernatant was then separated and used for the estimation of hydrogen peroxide (H_2_O_2_) and malondialdehyde (MDA) contents.

To determine the H_2_O_2_ content, the extract (0.5 mL) was mixed with potassium phosphate buffer (pH 7, 0.5 mL) and 1 M potassium iodide (1 mL) and incubated for 10 min. The absorbance was recorded at 390 nm and H_2_O_2_ content was calculated according to^53^ method.

For MDA content, the similar extract (0.5 mL) was mixed with 20% TCA (0.5 mL) in a capped test tubes containing 2- thiobarbituric acid (0.5%) and incubated in hot water bath at 95 oC for 30 min. Samples were then placed on ice bath to terminate the reaction and centrifuged at 12,000 × g for 10 min. Absorbance was recorded at 532, 600 and 450 nm^54^.

To determine the electrolyte leakage (EL), fresh leaf sample (0.5 g) was placed in distilled water (10 mL) and initial readings (EC_1_) were calculated by an Electric Conductivity Meter. Samples were then capped and incubated for 30 min in a boiling water bath, and then final reading (EC_2_) was measured. The percent EL was calculated as described by^55^ method.

### Estimation of total soluble proteins

Total soluble proteins were estimated by the method of^56^ using Coomassie brilliant blue. Plant extract (40 µL) was added in Bradford reagent (2.0 mL) and absorbance was recorded and protein was estimated using bovine serum albumin (BSA) as standards.

### Determination of antioxidant enzyme activities

Fresh leaf material (0.5 g) was crushed in liquid nitrogen with 5 mL (50 mM) potassium phosphate and centrifuged (20,000 rpm) at 4 °C. Supernatant was recovered and used for enzyme activities.

### Superoxide dismutase activity (SOD)

Potassium phosphate buffer (50 Mm, pH7.8, 100 mL) was mixed with L-methionine (201.34 Mm, 1.5 mL), NBT (1.76 mM, 1 mL), and Triton X-100 (0.75 mL) in a dark bottle and mark as reagent A. Riboflavin was used a reagent B. The reaction mixture contained 1 mL reagent A, 40 µL enzyme extract and 10 µL reagent B. Tubes were then placed in a dark aluminum foil folded box for 7 min for control and other set under aluminum foil lined box containing 2 fluorescent tubes of 20 W. After reaction completion the absorbance was recorded at 560 nm. The SOD activity was calculated as described by^57^ method.

### Catalase activity (CAT)

Enzyme extract (50 µL) was mixed with potassium phosphate buffer (50 mM) and H_2_O_2_ (15 mM). The initial absorbance of the mixture was recorded immediately and the decrease in absorbance was recorded after 1 min. The CAT activity was calculated as described by^58^ method.

### Ascorbate peroxidase activity (APX)

Reaction mixture was prepared containing 50 mM phosphate buffer (pH 7), 0.55 mM ascorbic acid and 0.1 mM H_2_O_2_, under dark conditions. Enzyme extract (50 µL) was mixed with reaction mixture and immediately recorded the change in absorbance for 20 min at 290 nm. The APX activity was calculated as described by^59^ method.

### Guaiacol peroxidase activity (GPX)

Reaction mixture containing potassium phosphate buffer (50 mM, pH7) was mixed with 19.4 µL of H_2_O_2_ (35%) and guaiacol (33 µL). the volume of mixture was adjusted up to 100 mL with potassium phosphate buffer. Change in absorbance was noted at 470 nm till 1 min. The GPX activity was calculated as described by^60^ method.

### Estimation of ascorbic acid

Ascorbic acid was estimated by adding plant samples (TCA extract, 500 µL), 2,6–dichlorophenolindophenol (DCPIP, 500 µL) and water (250 mL). The TCA extract of 500 µL was assorted in 500 µL of DCPIP and 250 µL water. The absorbance was measured at 600 nm against TCA as blank^61^.

### Estimation of glutathione

Plant extracts (TCA extract, 0.5 mL) were mixed with H_2_O (500 µL), 0.2 M phosphate buffer (pH 7.0, 500 µL) and 50 mM 5, 5-dithiobis (2-nitrobenzoic acid) (DTNB 100 µL) and incubated for 30 min. Absorbance was then recorded at 412 nm and amount was calculated against standard curve prepared by glutathione (GSH) as standard^61^.

### Antioxidant capacity of plant extracts

#### DPPH radical scavenging activity

Antioxidant capacity using 2,2-Diphenyl-1-picrylhydrazyl (DPPH) radical was performed by^62^ method. DPPH radical solution (100 μM) was mixed with methanolic extract in a similar proportion (500 µl each) and incubated under dark for 20 min. Absorbance was then recorded at 515 nm against solvent blank and percent inhibition was calculated by the following formula:

$${\text{I }}\% \, = {\text{ Abs}}_{{{\text{control}}}} - {\text{ Abs}}_{{{\text{sample}}}} /{\text{ Abs}}_{{{\text{control}}}} \times { 1}00$$

#### ABTS radical scavenging activity

Antioxidant capacity using 2,2′-azinobis(3-ethylbenzothiazoline-6-sulfonic acid (ABTS) radical was performed^63^. Methanolic extract (150 μL) was mixed with diluted ABTS reagent (7 mM ABTS and 2.45 mM potassium persulfate, 500 μL) and incubated in dark for 10 min. Absorbance of reaction mixture was recorded at 734 nm by Spectrophotometer (Jenway 3300, UK) and percent inhibition was calculated by the following formula:

$${\text{I }}\% \, = {\text{ Abs}}_{{{\text{control}}}} - {\text{ Abs}}_{{{\text{sample}}}} /{\text{ Abs}}_{{{\text{control}}}} \times { 1}00$$

#### Ferric reducing antioxidant power assay (FRAP)

FRAP reagent was made by adding acetate buffer (300 mM, pH 3.6), TPTZ (10 Mm) in HCl (40 Mm), and ferric chloride (0.054 g, 20 mM). Methanolic extract was mixed with 1 mL FRAP reagent and absorbance was recorded at 593 nm after 10 min of incubation^64^.

#### Total antioxidant capacity using phosphomolybdenum method (TAC)

TAC reagent was prepared by adding sulfuric acid (0.6 M), ammonium molybdate tetrahydrate (4 mM) and sodium phosphate dibasic solution (28 mM) in a ratio of 1:1:1^65^. Methanolic extract (100 µL) was mixed with TAC reagent (1 mL) and boiled for 90 min. After cooling, the absorbance of samples was measured at 765 nm.

#### Estimation of total phenolic contents

Air dried leaf samples (0.5 g) were homogenized in 10 mL 80% MeOH and incubated in a shaking water bath for 12 h. Extracts were then centrifuged at 4000 rmp and supernatant was collected for analysis.

Total phenolic content was estimated by Folin-Ciocalteu reagent method^66^. Plant extract was mixed with Folin-Ciocalteu reagent. After 5 min, saturated sodium carbonate solution (7.5%) was then added to the reaction mixture and incubated for 90 min. The absorbance was recorded at 765 nm and phenolic content was estimated using gallic acid as standard.

#### Estimation of total flavonoid content

Total flavonoid content was determined by^67^ method. Briefly, 10% aluminum chloride (50 µL), potassium acetate (50 µL) and distilled water (1.4 mL) were added in methanolic extracts (250 µL) and incubated for 40 min. Absorbance was then recorded at 415 nm and flavonoid content was estimated using Quercetin as standard.

### Statistical analyses

Data is presented in terms of mean ± standard error values of three biological replicates along with 06 technical replicates of each treatment. Analysis of variance and post-hoc test were performed to get significant differences among treatment means. SPSS (version 14) was used for all statistical analyses and graphs were plotted with the help of Sigma Plot (version 12.5).

### Ethics approval and consent to participate

The experimental research, and collection of plant material of this study complies with the relevant institutional, national, and international guidelines and legislation. Moringa seeds for experimentation were collected with permission.

## Results

### Vegetative growth

**Figure 1:**
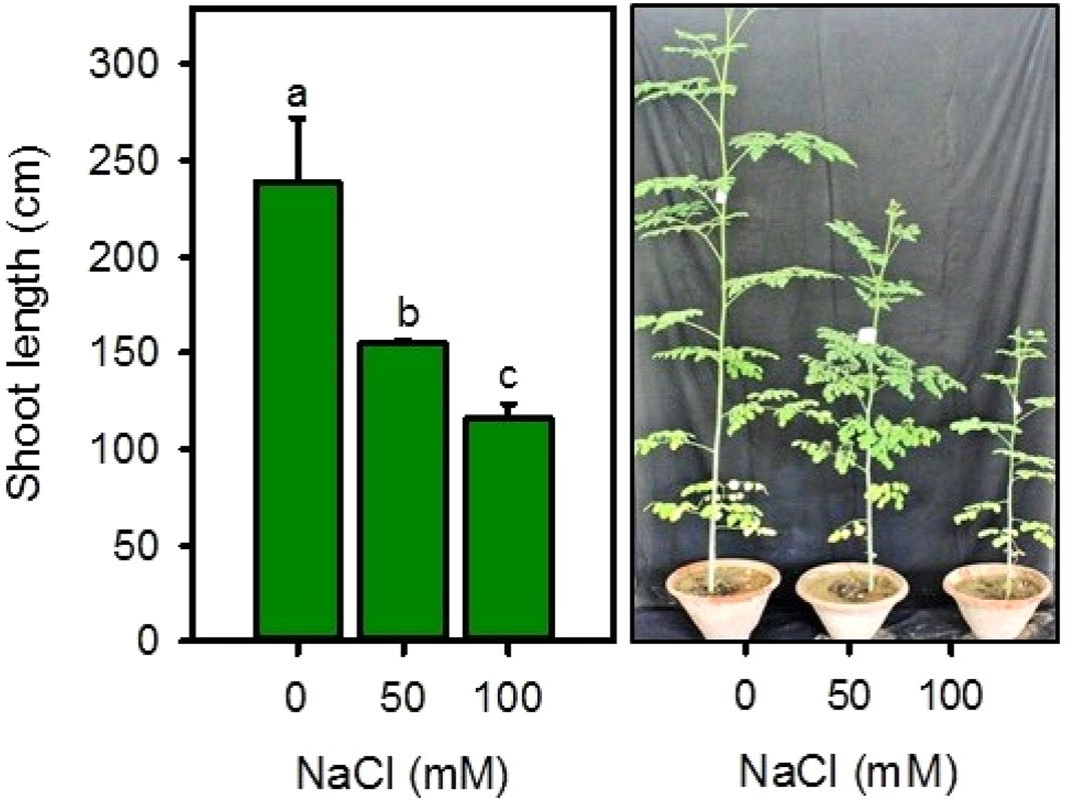
Effect of salinity on shoot length of *Moringa oleifera* after 40 days of treatment. Different letters represent significant differences at *P* < 0.05.

**Figure 2:**
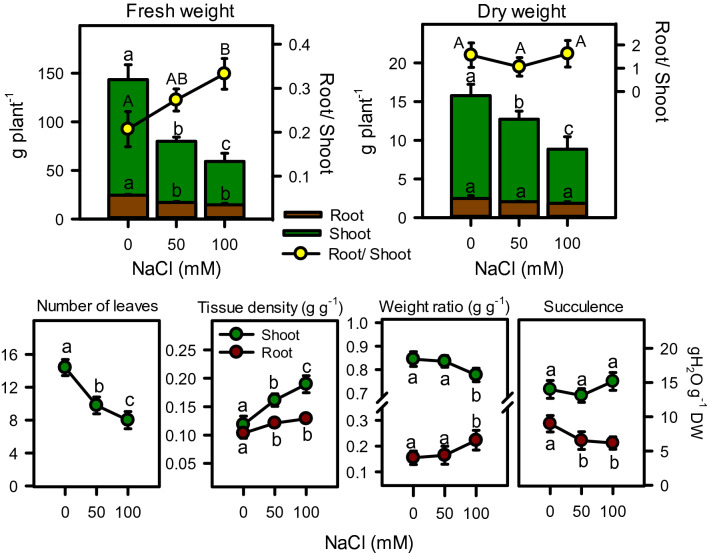
Fresh and dry biomass (shoot, root, and total plant), root/shoot, number of leaves, tissue density, weight ratios, and succulence of *Moringa oleifera* under different salinity treatments. Different letters indicate significant differences at *P* < 0.05.

Salinity stress significantly (*P* < 0.001) declined plant height and shoot biomass (fresh and dry), while it had no effect on root biomass and root/shoot ratio (Fig. 1, 2). Although salinity reduce shoot biomass, but the reduction in dry weight (19–40%) was much lesser than the fresh weight (47–63%) under both salinities, as compare to controls (Fig. 2). Root fresh weight was slightly declined () but root dry weight showed no effect of salt treatments than non-saline controls. Similarly, root/ shoot ratio for fresh biomass was increased with increasing salinity, while the ratio was unchanged for dry biomass (Fig. 2). Overall plant response was negatively correlated with salinity (r^2^ = − 0.997).

**Figure 3:**
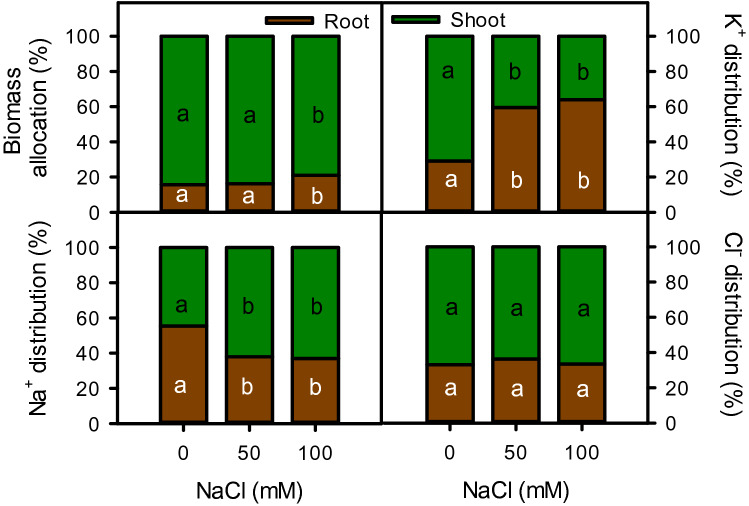
Biomass allocation and Na^+^ and Cl^−^ distribution in root and shoot of *Moringa oleifera* under different salinity treatments. Different letters indicate significant differences at *P* < 0.05.

Number of leaves, shoot weight ratio and root succulence were also decreased with increasing salinity, whereas tissue density of both shoot (r^2^ = 0.992) and root (r^2^ = 0.973) as well as root weight ratio (r^2^ = 0.922) were increased under salinity (Fig. 2). Increase in tissue density of shoot was more pronounced as compared to root, while weight ratios of both shoot and root were reduced only at high salinity (Fig. 2). The shoot became more succulent than root and remained intact under salinity. Root succulence initially decreased (27%) with onset of salinity but maintained with further increase in salinity. Biomass of plant allocation between root and shoot was unaffected by moderate salinity, however under high salinity the plant tend to allocate more biomass towards root than shoot (Fig. 3).

### Leaf pigments

**Figure 4:**
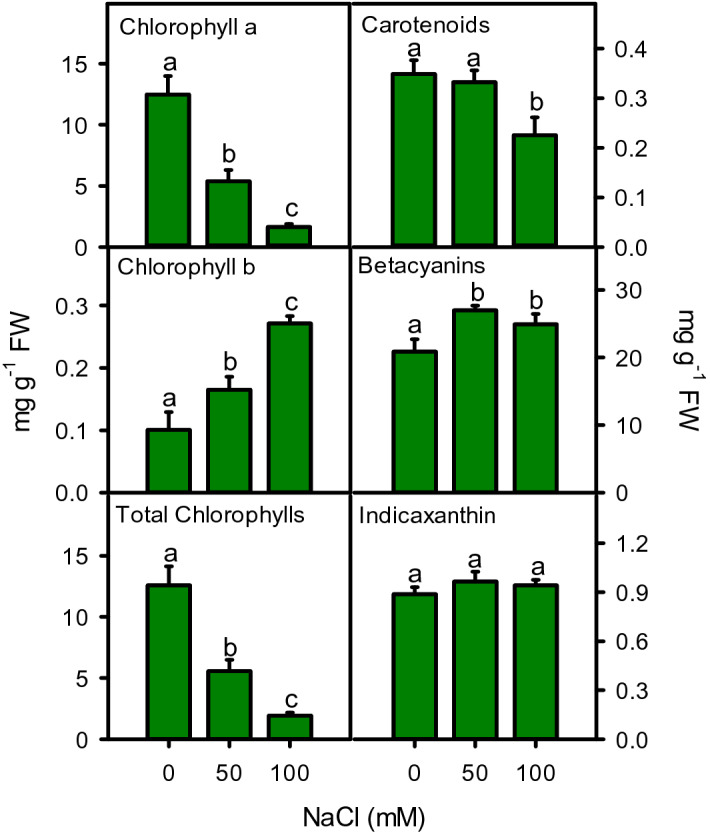
Pigments including chlorophyll *a*, *b*, total chlorophylls, carotenoids, betacyanins, and indicaxanthin of *Moringa oleifera* leaves under different salinity treatments. Different letters indicate significant differences at *P* < 0.05.

Leaf pigments were significantly (*P* < 0.001) affected by increasing salinity (Fig. 4). A substantial decrease in chlorophyll *a* (r^2^ = − 0.984) and total chlorophylls (r^2^ = − 0.983), while linear increase in chlorophyll *b* (r^2^ = 0.990) was recorded under salinity (Fig. 4). Carotenoids were decreased only in high salinity while, betacyanin were increased by both salinity treatments. Indicaxanthin were remain unchanged throughout the experiment (Fig. 4).

### Ions

**Figure 5:**
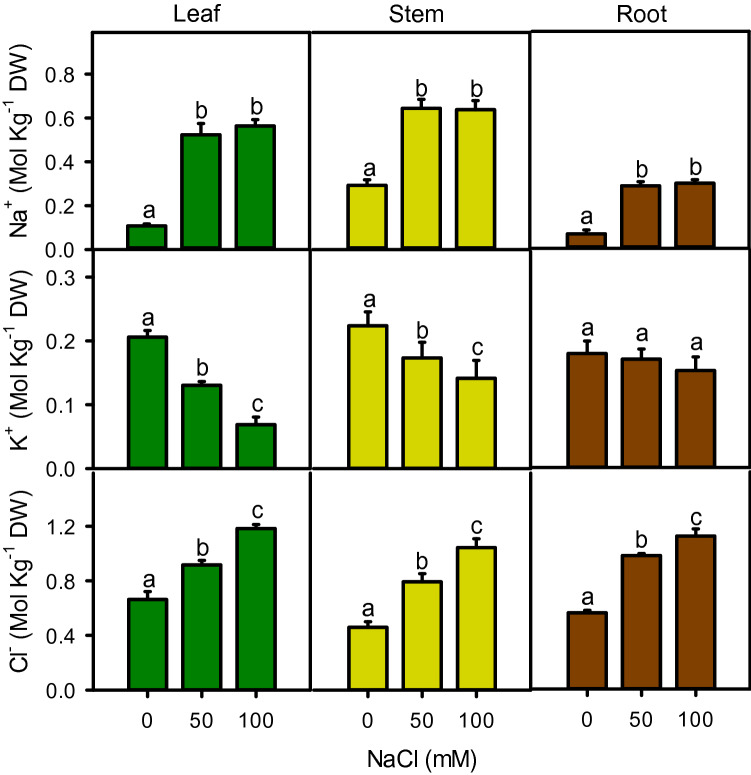
Contents of Na^+^, K^+^, and Cl^−^ in different parts of *Moringa* under different salinity treatments. Different letters indicate significant differences at *P* < 0.05.

Salinity significantly affected the cations (Na^+^ and K^+^) and anion (Cl^−^) accumulation with varying concentrations in leaf, stem, and root (Fig. 5). Generally, in all plant parts, Na^+^ content was sharply (*P* < 0.001) increased with the onset of salinity but maintained with further increase in salinity (Fig. 5). Leaf and stem accumulated more Na^+^ than root. However, K^+^ content of leaf (37–67%, r^2^ = − 0.998) and stem (23–37%, r^2^ =− 0.991) decreased linearly with increasing salinity while, root K^+^ was unaffected by salinity treatments. On contrary, Cl^−^ contents of all plant parts increased linearly with increasing salinity and the amount of Cl^−^ was higher than Na^+^.

### Osmotic adjustments

**Figure 6:**
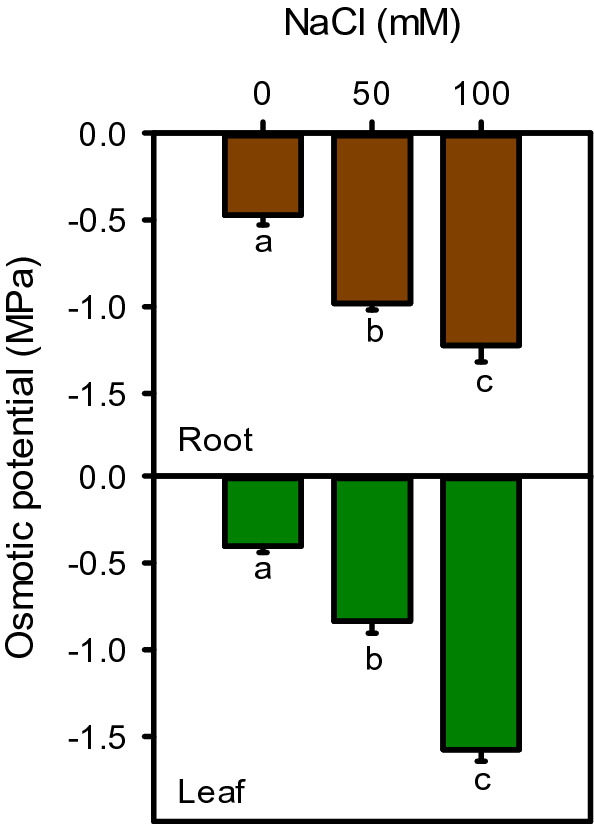
Osmotic potential of root and leaf of *Moringa oleifera* under different salinity treatments. Different letters indicate significant differences at *P* < 0.05.

Osmotic potentials (OP) of both leaf (107–158%) and root (107–290%) decreased significantly (*P* < 0.001) with increasing salinity (Fig. 6). Osmotic potential is positively correlated with plant fresh (r^2^ = 0.996) and dry biomass (r^2^ = 0.963), while negatively correlated with tissue density (r^2^ = − 0.996), succulence (r^2^ = − 0.390) and Cl^−^ content (r^2^ = − 0.976).

**Figure 7:**
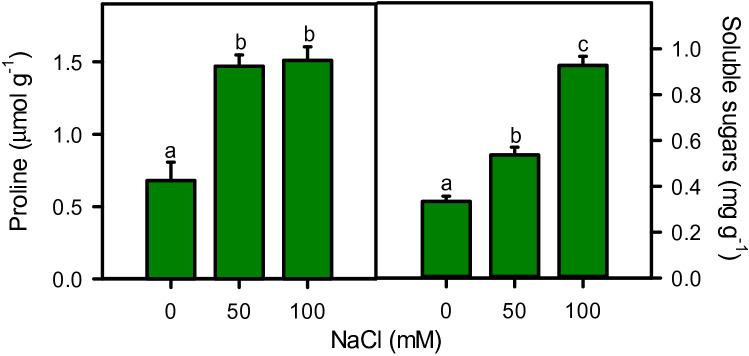
Proline and total soluble sugars of *Moringa oleifera* leaf under different salinity treatments. Different letters indicate significant differences at *P* < 0.05.

Proline (r^2^ = 0.886) and soluble sugar (r^2^ = 0.983) contents increased significantly under salinity stress (Fig. 7). Moringa sharply increase leaf proline with the onset of salinity (116%) which was statistically maintained at higher salinity (122%), as compared to control. While, soluble sugars increased gradually under moderate (61%) and high (177%) salinity, as compared to control (Fig. 7).

### Hydrogen peroxide, malondialdehyde and electrolyte leakage

**Figure 8:**
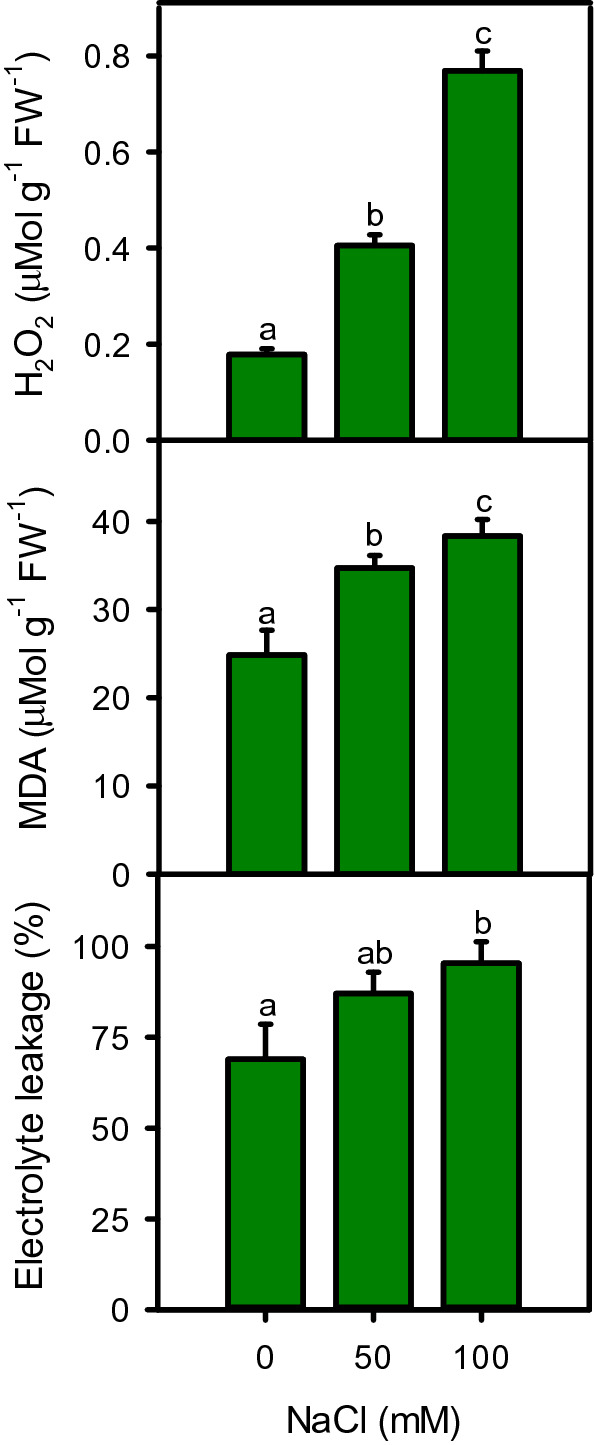
Damage markers including H_2_O_2_, MDA, and electrolyte leakage of *Moringa oleifera* under different salinity treatments. Different letters indicate significant differences at *P* < 0.05.

Oxidative stress markers including H_2_O_2_ (r^2^ = 0.991), MDA (r^2^ = 0.966) and EL (r^2^ = 0.977) were increased under salinity and their highest amount was found at 100 mM NaCl (Fig. 8). Both H_2_O_2_ (127–331%) and MDA (40–54%) contents were significantly increased under both salinity treatments, while EL increased significantly only at high salinity (38%), as compared to control. These markers including H_2_O_2_, MDA and EL showed positive correlation with leaf Na^+^ (r^2^ = 0.838, 0.983 and 0.973), and Cl^−^ (r^2^ = 0.993, 0.974 and 0.962), while negative correlation with plant fresh (r^2^ = − 0.912, − 0.996 and − 0.999) and dry biomass (r^2^ = − 0.997, − 0.961 and − 0.946).

### Antioxidant defense system

**Figure 9:**
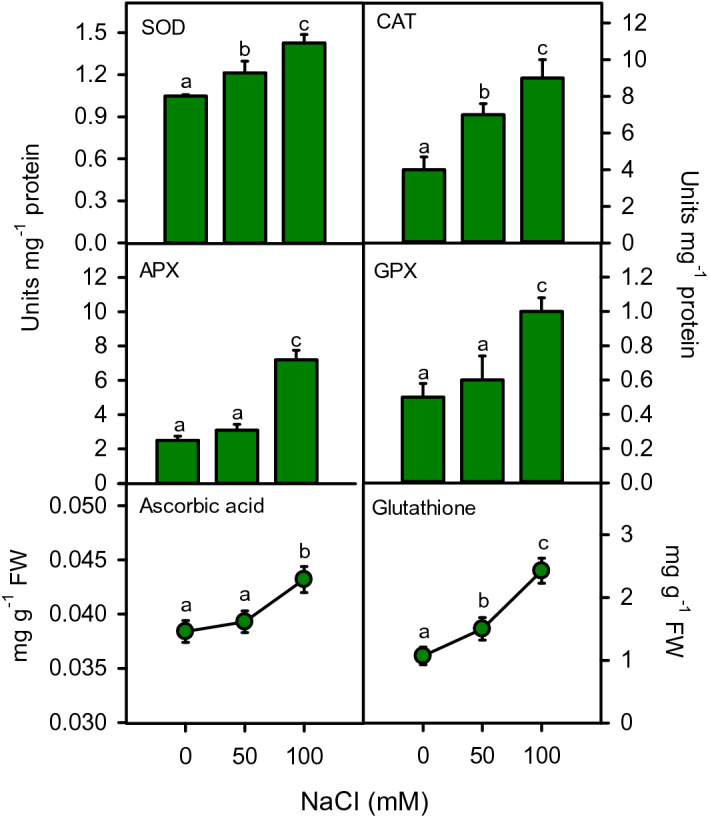
Activities of antioxidant enzyme including SOD, CAT, APX and GPX and contents of ascorbic acid and glutathione in *Moringa oleifera* under different salinity treatments. Different letters indicating significant differences at *P* < 0.05.

**Figure 10:**
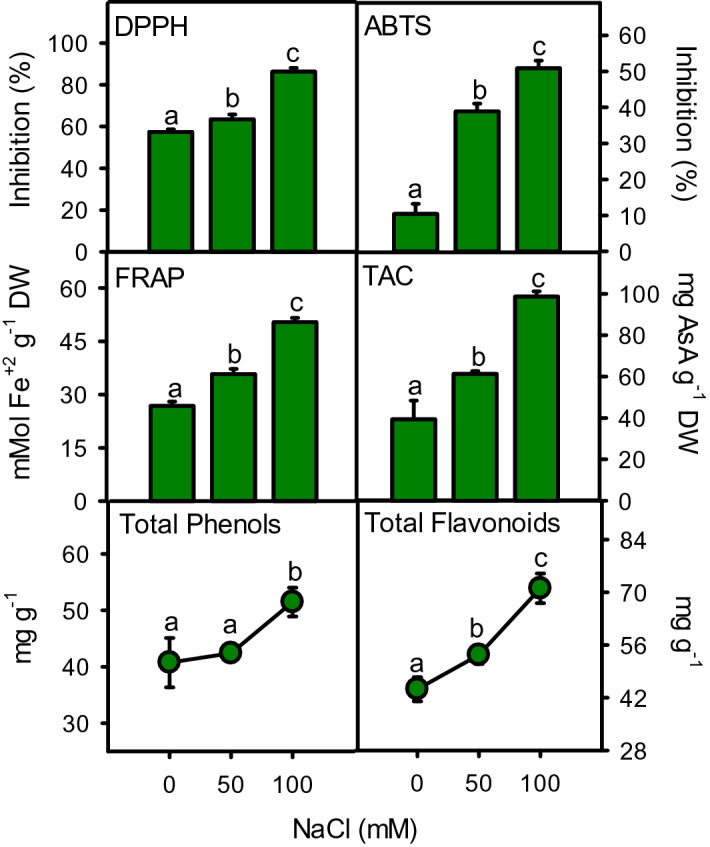
Antioxidant capacity (DPPH, ABTS, FRAP and TAC), total phenols and flavonoid contents in *Moringa oleifera* under different salinity treatments. Different letters indicate significant differences at *P* < 0.05.

**Figure 11:**
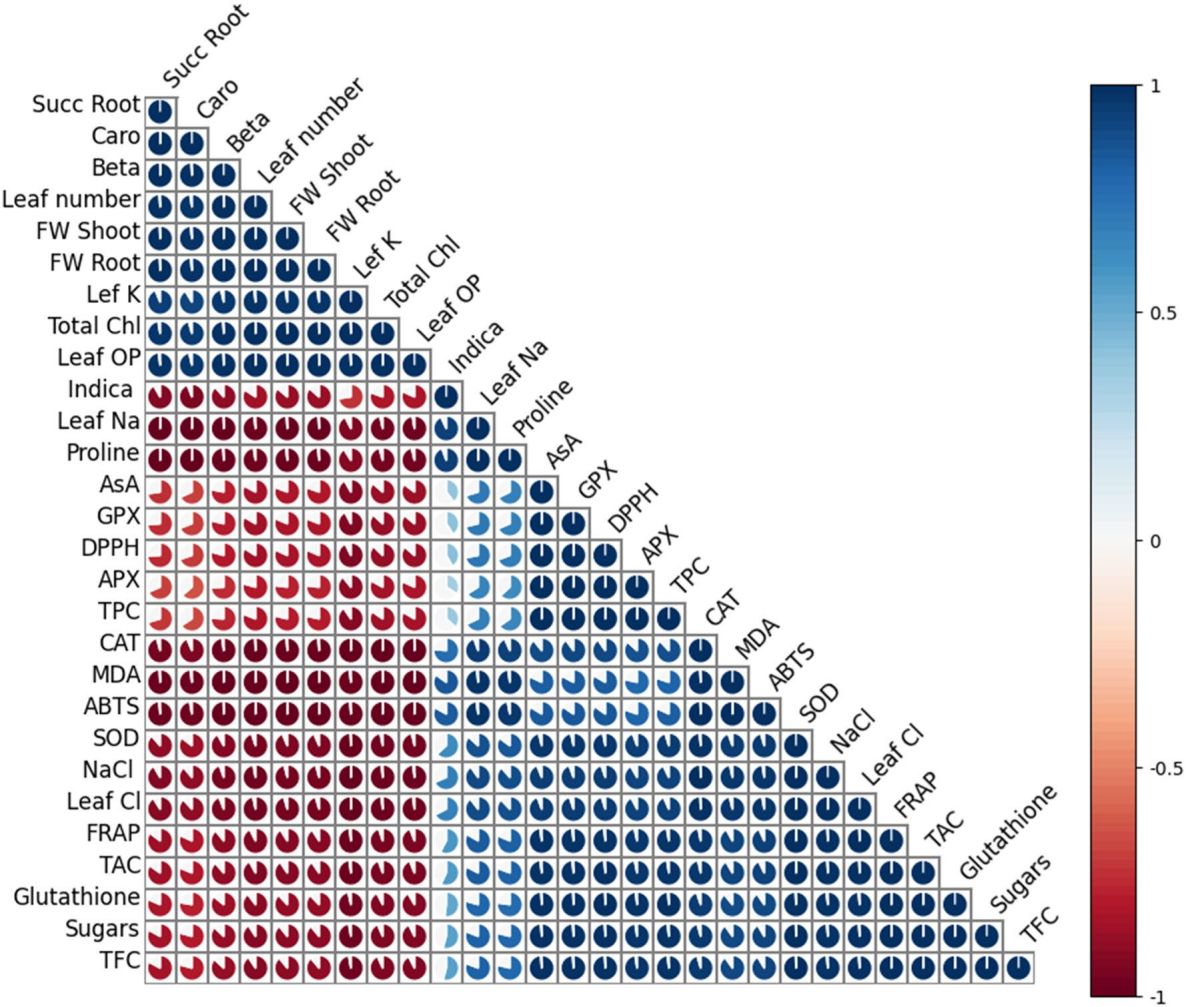
Heat map representing Pearson’s correlation among different traits of *Moringa Oleifera* by using R-software version 4.2.2.

Antioxidant defense system including antioxidant enzymes (SOD, CAT, APX, and GPX), substrates (ascorbic acid and glutathione), antioxidant capacity (DPPH, FRAP, ABTS, TAC), and antioxidant compounds (total phenols and flavonoids) showed remarkable increase under salinity stress (Figs. 9, 10). SOD, CAT, and glutathione increased (*P* < 0.001) linearly at moderate (16, 75, 40%) and high (36, 125, and 127%) salinities. Whereas, APX (188%), GPX (100%) and ascorbic acid (13%) increased only at high salinity (Fig. 9). Antioxidant capacity measured by all testing systems enhanced substantially under salinity (Fig. 10). The increase in DPPH, ABTS, FRAP, and TAC was 11%, 273%, 33% and 55% at moderate salinity, while 50%, 388%, 88%, and 150% at high salinity, as compared to control. Total phenols were maintained at moderate and significantly enhanced at high (26%) salinity. However, total flavonoids increased (21–60%) gradually with increasing salinity (Fig. 10). All antioxidant defense parameters showed strong positive correlations with increasing salinity, accumulation of toxic ions (Na^+^ and Cl^−^) and damage markers (H_2_O_2_, MDA and EL), while negative correlation with plant growth parameters (Fig. 11).

## Discussion

Plants invest sufficient energy in various metabolic processes to deal with environmental stresses. It has been reported that moringa withstands salinity stress^68–70^, however its tolerance mechanism and eco-physiological responses were not well documented. This study was designed to investigate the effect of salt stress on growth, leaf pigments, ion accumulation, osmotic adjustment, and antioxidant defense system involved in salinity tolerance of the plant. In addition, the medicinal potential of salt stressed moringa in terms of the content and activity of antioxidant compounds was also evaluated.

Salt stress negatively correlated with growth parameters such as plant height, accumulation and allocation of fresh and dry biomass, and number of leaves (Fig. 1, 2), which was in line with the previously reported data^71,72^. In contrast, salinity increased the root/ shoot fresh mass ratio, tissue density, and biomass allocation to root. Moringa effectively cope-with 50 mM NaCl by maintaining succulence, weight ratios, and biomass allocation patterns of both shoot and root with non (root) to minimal (shoot) reduction (19%) in dry biomass. However, 100 mM NaCl remarkably declined all growth parameters. Generally, plant needs half of its energy to regulate metabolic processes, and the rest for growth and standing biomass^73^. Beside reducing gross energy production through photosynthesis, salt stress diverts ample amount of energy into stress regulation mechanisms rather using it for growth, as found in this study. Plant root is the first organ to encounter salt stress, hence root growth is particularly sensitive to root-zone salinity^74^. Before causing ion toxicity, high salt content in the rooting medium limits water availability, causing a physiological drought and reduction tissue water content. In this study, although high salinity reduced root fresh weight, but it still tend to allocate more biomass to the root. This could be a plant’s strategy to adjust under salinity by allocating new biomass to the tissues that get the most limiting resources^75^ as found in cotton, wheat, and sunflower^76–78^. In addition, the development of roots is crucial for maintaining osmotic balance and ion homeostasis under salt stress. Accumulation and alteration in root and shoot biomass ratio quantifies the number of resources allocated to leaf carbon assimilation relative to water and nutrient absorption, thus it is an important indicator of the functional balance between these processes^15^. The change in the proportion of dry biomass (from 84 to 79% for the shoot and 16% to 21% for root) and increasing root/ shoot fresh mass ratio indicates the ability of the plant to manage water and mineral uptake, especially at moderate salinity.

Salinity exposure lowers the photosynthetic activities and leaf pigments by reducing leaf area in many plants. Total chlorophylls were significantly reduced with increasing salinity (r^2^ = − 0.983). The decrease in total photosynthetic area and chlorophyll content suggests a strategy to protect photosynthetic apparatus from salt-induced photo-damage and avoid the excessive generation of ROS, especially at moderate salinity^31^ also reported similar pattern of pigment reduction in moringa upon salt exposure. The decrease in total chlorophylls might be due to the disruption in photosynthetic machinery, malfunctioning of pigments, instability of pigment-protein complexes, and structural damages in the light-harvesting complex^79^. In addition, high salt content leads to ROS generation in chloroplasts that break the double bonds of unsaturated fatty acids, thereby damaging chloroplast membrane and resulting in chlorophyll leakage from the thylakoids^80^. Due to its photodynamic action, free chlorophylls must be degraded quickly to prevent cellular damage^81^. In addition, enhanced chlorophyllase activity and salt-induced reduction in light-harvesting apparatus could be the reason behind reduced photosynthetic efficiency. Moringa can also lower the electron transport and photosynthetic rate to avoid salt induced photo-oxidation and oxidative stress^70^.

Other leaf pigments such as carotenoids, betacyanins, and indicaxanthin are also involved in mitigating the harmful effects of salinity stress. These pigments are related to cell protection against salt induced photo-oxidative damage, which can declined the chlorophyll’s excitation pressure leading to reduced light-harvesting capability^33^. In the present study, the increasing content of betacyanins (significantly), indicaxanthin (non-significantly), and unchanged carotenoids indicates a better management of photo-damage and oxidative stress under moderate salinity. While, decreased carotenoids at high salinity exhibited a low capacity for absorbing and transferring light energy and less heat dissipation via the xanthophylls cycle^82^. The insufficient heat dissipation is associated with excessive generation of superoxide and other free radicals^83^. In this situation, betacyanin and indicaxanthin play their part to alleviate salt-induced oxidative damage^84^, thus their contents were increased in the plant. In addition, these compounds may serve as osmolytes or osmoprotectants to safeguard physiological processes by altering the pool of amino acids^85^.

Accumulation of toxic ions (Na^+^ and Cl^−^) in different plant parts reduced the growth and productivity of moringa plant under salt stress.^69,86^ also reported a linear increase in Na^+^ and a decrease in K^+^ contents in different parts of the plant with increasing salinity. Ion accumulation reinforce the osmotic potential gradient, which may help plant to absorb water and prevent drastic effects of physiological drought posed by salinity. However, excessive up take of Na^+^ and Cl^−^ by roots inhibited the availability of K^+^ (by Na r^2^ = − 0.926,by Cl r^2^ = − 0.997) and other essential minerals such as Ca^2+^, Mg^2+^, N, and P^86–88^. Interestingly, Na^+^ increased drastically in all parts of the plant only at the onset of salinity, while further increase in salinity had no effect on Na^+^ accumulation. When root-zone salinity increases, it substantially reduces transpiration, which may restrict the further Na^+^ uptake. In addition, moringa may re-translocate Na^+^ from above-ground parts to the roots as reported in other species^89,90^. This plant behaves like a salt includer species by accumulating a substantially higher amount of Na^+^ in above-ground parts than roots. Increasing root-zone salinity disturbs the root/shoot distribution of both Na^+^ and K^+^. The proportion of Na^+^ increased substantially in the shoot, while it decreased in the root, whereas, for K^+^, the situation was antipodal under salinity. An increased root/shoot K^+^ distribution may help the plant to maintain root turgor by using K^+^ as cheap osmoticum. On the other hand, above-ground plant parts possibly suffer K^+^ starvation due to the limited K^+^ transport from root to shoot, especially at high salinity. Potassium is an essential nutrient for plant growth under stressful conditions. In this study, decreased K^+^ uptake due to high Na^+^ influx (r^2^ = − 0.926) might lead to ion competition on K^+^ transporter over nonselective cation channel. This situation may create membrane depolarization and plasma membrane disintegration, which displace essential minerals (such as K^+^, Ca^2+^, Mg^2+^, etc.) and water^68^. On the contrary, the root bulk of Na^+^ was not much increased under salinity as compared to above-ground parts, while K^+^ content was maintained in all salinities. Plant generally take up further K^+^ and limits Na^+^ absorption in roots, while seizing the Na^+^ loading into the xylem stream, which ultimately minimize the net Na^+^ influx to different parts of plants^15^, as found in other plants^91,92^.

In moringa, the uptake and transport of Cl^−^ are different than Na^+^. The accumulation of Cl^−^ was almost 2 × higher in leaf and stem, while 4 × in the root, as compared to Na^+^. Similar results were found, when the plant grown up to 16 dSm^−1^ NaCl^86^. In contrast to Na^+^, the amount of Cl^−^ increased linearly with increasing salinity with almost similar content in all plant parts. The unchanged root/shoot Cl^−^ distribution, especially at 50 mM NaCl, could be beneficial for maintaining turgor potential^93^. Chloride also acts as counter charge ion against the rising cationic concentration (Na^+^, K^+^, etc.), which may help to reduce the membrane potential as well as work as an osmolytes under saline conditions^94^. It also helps in pH regulation by augmenting the H^+^-ATPase activity. However, higher Cl^−^ concentration also affects the dipole moment of lipid bilayers, hinders the interchange of essential minerals, metabolites and toxins, and perhaps slowdown metabolic processes. In addition, it could interfere NO^3^ uptake and restrict nitrogen metabolism, which reduce plant growth, as evident in our results.

Osmotic potential gradient and maintenance of leaf succulence are considered essential determinants for the growth of most dicotyledonous plants under salt stress^95^. Osmotic potentials (OP) of both root and leaf showed a linear decline due to increasing salinity (r^2^ = − 0.979). This indicates an osmoconformor strategy of moringa, as reported in other plants^74^. Decrease in OP requires excessive solute uptake, to create sufficient turgor for cell growth and elongation^15,96^. No change in leaf succulence indicate the water-conserving strategy of the plant that helps in retaining cell wall integrity and maintaining growth, especially under moderate salinity^97,98^. To achieve workable OP, most plants accumulate ions as cheap osmoticum^99^, because synthesizing organic osmolytes require a high cost of energy^100,101^. The more negative OP could be due to accumulation of Na^+^, K^+^, and Cl^−^ along with organic osmolytes such as soluble sugars and proline.

Results showed a high amount of proline and liner increase in soluble sugar contents in leaves, as reported earlier in *M. olifera*^72^ and its sister species *M. peregrina*^102,103^. Organic osmsolyte accumulation effectively contributed in osmotic adjustments (proline r^2^ = − 0.979 and soluble sugar r^2^ = − 0.979), maintaining turgidity and protection of cellular metabolism from salt toxicity^15^. Accumulation of both proline and soluble sugar contents indicates their active involvement in osmotic adjustment and salt tolerance of moringa, as reported in different plant species^104–107^. These compounds protect and stabilize enzymes and proteins, reduce oxidation of lipid bilayers, work as free radical scavengers, and cell redox balancers, provide sites for carbon and nitrogen storage, and are involved in cytosolic pH regulation^108^. Additionally, these compounds engaged in stress signaling and modulating gene expression under stressful conditions^109,110^.

High salinity stress disrupts the electron transport chain leading to oxidative damages in plants. The excessive energy generated during electrochemical reactions can be dissipated through the Mehler reaction, leading to the overproduction of ROS (like H_2_O_2_). Damage markers including H_2_O_2_, EL, and MDA are linked with the series of free radical generation reactions can damage cellular structures and macromolecules, imbalance the cellular redox potential, and decrease membrane fluidity, leading to electrolyte leakages and rapid desiccation. Membranes are most sensitive to oxidative stress and represent a suitable stress tolerance marker. H_2_O_2_ works as a signaling molecule during stress tolerance mechanisms, however its higher levels inactivate many antioxidative and Calvin cycle enzymes and is directly associated with membrane and pigment damages, which in turn over-reduce photosynthetic machinery that generate further radicals^111,112^. In this study, moringa presented both examples of low and high levels of H_2_O_2_ with contrasting effects. At 50 mM NaCl, it appears that H_2_O_2_ has been used for the perceiving and managing the salt stress which helped in activating antioxidant enzymes (SOD and CAT) and molecules (glutathione and flavonoids) with high antioxidant capacity (DPPH, ABTS, FRAP, and TAC). At this salinity, a significantly unchanged membrane fluidity (EL) and higher levels of stress adopter molecules (proline and soluble sugars) with minimal loss in plant dry weight indicating a positive aspect of H_2_O_2_ in salt tolerance of the plant. In addition, a decrease in chlorophyll content suggests an adaptive strategy to avoid absorption of excessive light, thus, restricting ROS concentration under workable limits. However, when salt stress intensified to 100 mM NaCl, the cytotoxic levels of Na^+^ and Cl^−^ generated a burst of ROS that exceeded the plants manageable threshold limit, causing damage to cellular structures and membranes as reflected by elevated MDA and EL levels. This could be a reason for stunted growth and substantial reduction in biomass of the plant under high salinity.

Activities of antioxidant enzymes including SOD (r^2^ = 0.997), APX (r^2^ = 0.918), CAT (r^2^ = 0.993) and GPX (r^2^ = 0.944) increased significantly, when the plant underwent salinity stress, as reported earlier^103^. As the first line of defense, SOD converts toxic superoxide radical (O_2_^*−^) into H_2_O_2_ at chloroplast level, which further detoxifies into water with the help of other peroxidases. In chloroplast, generally, APX utilizes ascorbate to convert H_2_O_2_ into water, while CAT and GPX generally works in the cytoplasm. In moringa*,* the Asada–Halliwell pathway enzymes were upregulated in an organized way, contributing to enhanced protection against ROS, as found in other plants^113,114^. Antioxidant enzyme activities of the plant increased progressively with rising ROS concentration^23^. In addition, increased contents of ascorbate and glutathione were also linked with direct ROS quenching and raised activities of APX and GPX^115^. At moderate salinity, increased SOD and CAT activities with unchanged levels of APX, GPX, and ascorbate suggest a balanced regulation of ROS with no significant membrane damage (unchanged EL). Here, moringa appeared to manage ROS concentration at both chloroplast and cytosol levels with minimal energy expenditure. In further, the unchanged APX but higher SOD and CAT activities suggest that H_2_O_2_ was produced at chloroplast but accumulated in the cytoplasm. Whereas, at higher salinity, a substantial increase in SOD, CAT, APX, and GPX activities indicate considerable ROS burst throughout the cell, demanding strong protection at the cellular and sub-cellular levels. Therefore, an ample amount of energy was required for oxidative stress management, which cost a drastic reduction in growth and biomass. The results of increased level of H_2_O_2_, MDA and EL and their strong correlation with increased antioxidant enzymes activities are in line with the antioxidative responses of other salt-tolerant plants^116,117^.

Moringa employed a well-discriminative defense system consisting of antioxidant enzymes as well as strong activities of antioxidant compounds to maintain a cellular redox balance. Antioxidant compounds like polyphenols (TPC) and flavonoids (TFC) are direct quenchers of free radicals, thus their quantity and composition varies according to the intensity of applied stress^118^. The level of TFC increased linearly with increasing salinity, while TPC was only increased at high salinity. Likewise, antioxidant capacity measured by DPPH, ABTS, FRAP, and TAC systems increased with increasing salinity, as reported earlier^72^. A slight increase in antioxidant compounds and their activity indicates a regulatory response at moderate salinity. In contrast, substantially higher content and activity of these compounds suggesting an apparent state of emergency to deal with oxidative burst under high salinity, which is also reflected in a drastic growth reduction. Both TPC (r^2^ = 0.929) and TFC (r^2^ = 0.983) showed a strong positive correlation with salinity, damage markers (r^2^ = 0.802 to 903), antioxidative enzymes (r^2^ = 0.9880 to 0.999) and substrates (r^2^ = 0.985 to 0.999), indicating their role in stress tolerance of moringa, as found in other species^119,120^. These compounds also protect photosynthetic machinery against photo-oxidation^121^, as light-harvesting complex is susceptible to oxidative damage. Moreover, these compounds provide shield against UV, high temperature, heat, and desiccation, which are often associated with salt stress. In this capacity, these compounds safeguard chloroplast and prevent further production of harmful singlet oxygen^122,123^. A variety of such phenolic compounds including gallic acid, catechin, vanillin, quercetin, kaempferol, naringin, and rutin and other antioxidants like ascorbic acid, carotene, and isothiocyanates, has been found in moringa^72,87,124,125^. A strong correlation of these compounds with effectively higher radical scavenging (DPPH and ABTS) and reducing power (FRAP and TAC) capacities indicating their major role in antioxidant defense of moringa, as found in *Suaeda monaica*, *Alhagi maurorum*, and *Dalbergia latifolia*^126,127^.

Besides their role in plant stress management, these natural antioxidants are high-value compounds for medicinal purposes. This plant is a well-known edible medicinal plant with various health benefits against widespread diseases and environmental toxins^128^. The level of antioxidant compounds of moringa found in this study was comparable to many antioxidant-rich plants and even higher than most of the glycophytes^129–132^. Moringa leaf extracts showed different levels of ROS protection studied by different *in-vitro* and *in-vivo* models^133,134^. Nevertheless, moringa extracts can be used for many other treatments including diabetes, hypertension, inflammation, cholesterol, bacterial and viral infections, and tumors^124,128,135–137^. It has been established that damage caused by free radicals have been linked to many of such diseases and as a treatment, antioxidants have shown potent effect against them. Therefore, increasing antioxidant activity and levels of bioactive phytochemicals could be used as a yardstick for increasing medicinal potential of moringa. At moderate salinity, the overall effect on the yield of bioactive compounds was almost 21% higher than control, with up to 3 folds higher antioxidant activity. It can be deduced that moringa grown on moderately saline soils could yield higher contents of high-value bioactive compounds with more robust antioxidant activity that can be used for domestic and industrial purposes. On the other hand, it can also be grown on high saline soils of up to 100 mM NaCl and produce even better quantities of bioactive compounds, but the considerable reduction in dry biomass (− 44%) cannot be ignored. For such conditions, studies for the growth improvement using different strategies/ techniques would be helpful to get sufficient biomass from theoretically unproductive soils. In addition, the results of this study are encouraging to study the effect of salinity on other reported and untapped medicinal properties of moringa and other medicinal plants.

## Data Availability

The data sets used and/or analyzed during the current study are available from the corresponding authors on reasonable request.
